# How Malaria Parasites Avoid Running Out of Ammo

**DOI:** 10.1371/journal.pgen.1004878

**Published:** 2014-12-18

**Authors:** David E. Arnot

**Affiliations:** University of Edinburgh, School of Biological Sciences, Institute for Immunology and Infection Research, Edinburgh, United Kingdom; Weill Medical College of Cornell University, United States of America

## Antigenic Variation in *Plasmodium falciparum*

Malaria parasites operate antigenic variation systems to avoid antibody recognition, thereby inhibiting their host's capacity to clear infections [1]. *Plasmodium falciparum*, the most pathogenic of the human malaria species, bases immune evasion on switching expression of approximately 60 *var* genes. These encode transmembrane proteins (PfEMP1 antigens) on the red blood cell surface, which allow intraerythrocytic (IE) parasites to adhere to endothelial molecules [2]. PfEMP1 proteins are thus not simply antigenic decoys, but functional vascular adhesion receptors. The disappearance of older parasites from the bloodstream results from PfEMP1 binding to endothelial receptors, a process called sequestration. This enables replicating stages to avoid the dangerous passage through the spleen, the main organ of defence against blood infection [3]. In this issue, Claessens et al. [4] present new and intriguing data on how antigenic diversity can be continuously generated during persistent malaria infections.

## Linking Antigenic Variation, Sequestration, and Pathology

Sequestration affects the course of clinical malaria [5]; and recent studies have linked the binding of specific PfEMP1s to endothelial protein C receptor (EPCR) with severe malaria pathology [6]. This extends studies connecting pregnancy malaria with binding of a form of PfEMP1 called VAR2CSA to placental chondroitin sulphate A (CSA) [7]. Individuals who experience multiple infections gradually acquire low level immunity that prevents the severe symptoms of the disease, but that does not prevent infection. One model of malaria pathogenesis proposes that after repeated exposures to parasites, there is progressive acquisition of blocking antibodies to a broad spectrum of PfEMP1 antigens. The most strongly adhesive PfEMP1 variants appear in early infections, since such variants would have greatest advantage in the absence of effective blocking antibodies. Naïve hosts would be most at risk, and the appearance of novel host receptors, for example the distinctive CSA present on the placental endothelial cells, selects for parasite PfEMP1 variants, which first time mothers would not previously have experienced and to which they had no antibodies [8].

## The Repertoire Problem

While the “PfEMP1 binding determines pathology” hypothesis offers explanations for several observations on severe malaria and the age-dependent acquisition of immunity, it is not just the details that remain to be nailed down. The PfEMP1 proteins are encoded by approximately 60 *var* genes, their extracellular portion encoded by exon 1, a smaller intracellular domain encoded by exon 2. The extracellular domains are highly ordered combinations of 628 “conserved minimal PfEMP1 building blocks” [9]. Practically all PfEMP1 encoding genes are intact and expressed in situ from telomeric and internal sites on 13 of the 14 *P. falciparum* chromosomes. This is a modest and conservatively ordered assembly compared to the approximately 1,600 strong trypanosome variant surface glycoprotein gene battery, most of which (65%) are pseudogenes on 11 megachromosomes, several intermediate chromosomes, and approximately 100 minichromosomes [10]. How *P. falciparum* modulates variant switching to avoid “running out of repertoire” during infections is not understood, particularly since switching rates appear high enough to easily run through 60 genes in an infection [11]. It is also notable that *P. falciparum* avoids creating *var* pseudogenes with trypanosome-like abandon.

## The Whole Genome Sequencing Approach

Understanding the generation of *var* gene diversity clearly requires closer study of *var* recombination; and significant advances are reported by Claessens et al. in this issue[4]. Studies of a handful of *var* crossovers revealed that it is usually ectopic (nonallelic) [12]–[16]. However, such small samples precluded crossover rate estimates and did not definitively establish where and when *var* recombination occurs. To increase event detection by screening large numbers of genomes, Claessens et al. [4] established cultures of *P. falciparum* isolates prior to cloning by limit dilution and re-expansion from single infected red blood cells. As numerous clonal lineages were generated, mutations arising in mitotically replicating cultures could be detected by whole genome sequencing (WGS). Remarkably, Claessens et al. [4]have now sequenced over 200 *P. falciparum* clone genomes.

**Figure 1:**
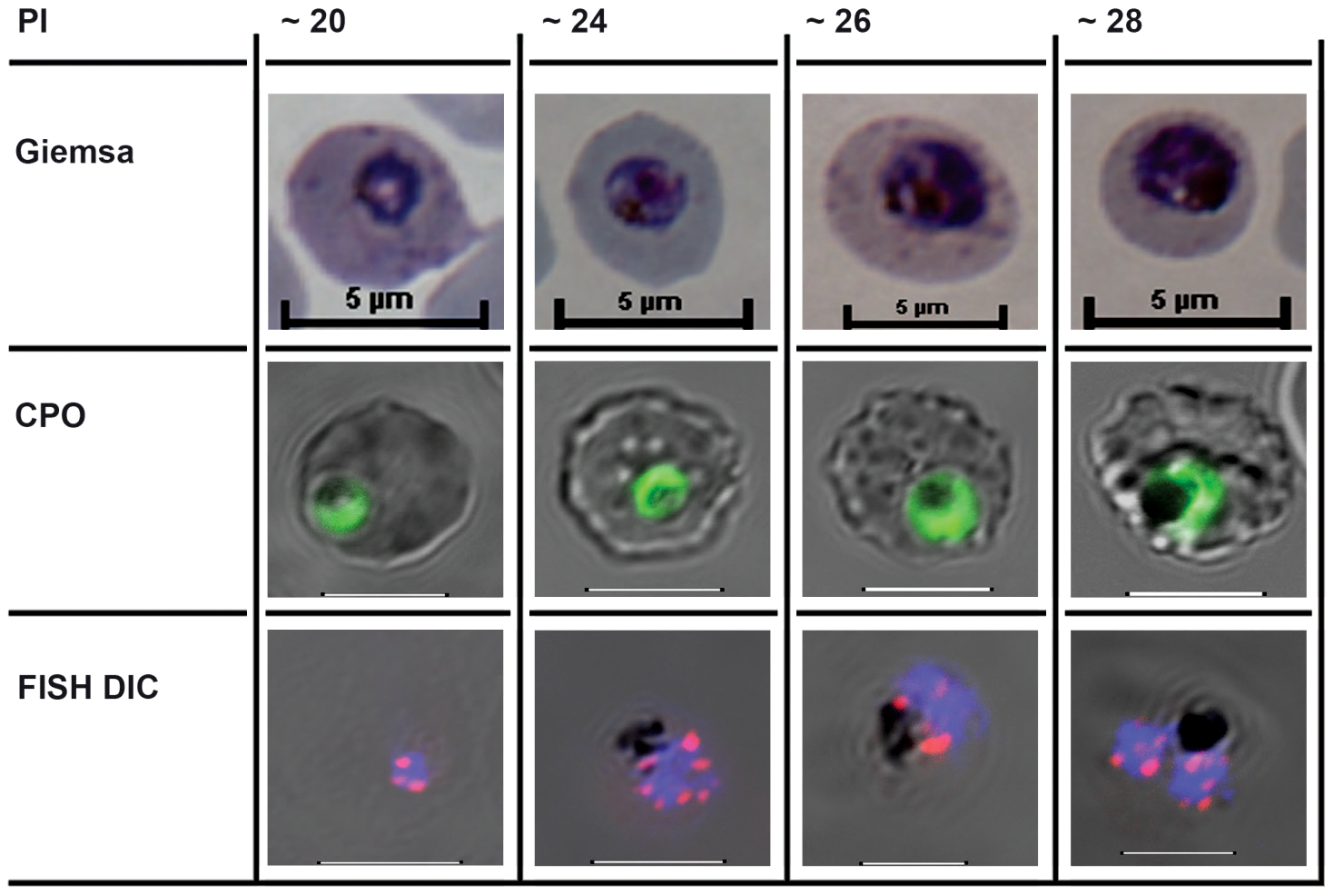
The cell biological context of *P. falciparum* intraerythrocytic (IE) mitotic recombination. The first mitotic division of single IE *P. falciparum* genomes occurs from around hours 20–28 of postmerozoite invasion (PI). The three rows illustrate different staining methods used to visualise parasites, nuclei, and chromosomes before, during, and immediately after the first of the parasite's asynchronous mitotic divisions [18]. Giemsa: the developing trophozoite visualised after methanol fixation and Giemsa staining. CPO: Fluorescent staining following Coriphosphine O (CPO) uptake by parasitized erythrocytes. CPO, an acidic acridine derivative, is membrane permeable but shows no fluorescence when free in red blood cells and gives strong green emission using the argon laser (488 nm) when bound to parasite DNA in intact IE. FISH Differential Interference Contrast (DIC): Fluorescence in situ hybridisation (FISH) using *cy3*-labelled *P. falciparum Rep 20* sequences, plus DAPI fluorescence, superimposed on the differential interference contrast image of intact IE parasites. Host cell membranes and vacuolar haemozoin are apparent. *Rep 20* hybridises to repetitive sequences at both telomeres of each of the 14 chromosomes. Telomere clustering is evident before, during, and after the nuclear division. It is noteworthy that *var* genes are often found in tandem arrays immediately adjacent to the telomere repeats, thus telomere clustering could potentially align them for more efficient recombination. The most likely mitotic recombination scenario would appear to be that both DNA secondary structure-induced double strand breaks and microhomology mediated *var* recombination-associated repair occur during the DNA replication process itself.

Analysis of 37 subclones of the 3D7 parent clone revealed 20 newly arising single nucleotide polymorphisms (SNPs) and 40 de novo structural genome changes—ten duplications, eight deletions, and 22 translocations. Strikingly, of the 19 structural changes that affected 3D7 exons, all recombined *var* genes. Other isolate analyses are less comprehensive, but WGS of other similarly generated clonal populations detected 11, 13, and zero *var* exon 1 recombinations in the Dd2, W2, and HB3 isolates, respectively. The WGS confirms earlier estimates of Bopp et al. [14] that the SNP mutation rates appear relatively constant between isolates (approximately 9×10^−3^ per replication cycle), and that genome rearrangements are highly concentrated in regions containing *var* genes. The *var* recombination to SNP ratio was calculated to be 0.25, 0.35, 0.54, and zero for 3D7, Dd2, W2, and HB3 respectively, the rate at which *var* genes recombine estimated to be 2×10^−3^ per replication cycle. In each 48 hour replication cycle, around 0.2% of parasites could contain a newly recombined *var* gene. Millions of new *var* genes will be created with every 48 hour asexual IE replication cycle, presumably when different *var* genes are in close proximity [13] during the mitotic chromosome divisions (Fig. 1).

## Recombination Breakpoints

WGS yielded an abundant harvest of over 100 crossovers to analyse. These new events conform to the pattern of highly structured ectopic recombination recently reported by Sander et al. [16]. Participating *var* genes are in the same orientation, often with multiple recombination breakpoints, yet generating functional recombinants by in-frame joining of sequences of the same domain type. Intrinsically recombinogenic DNA secondary structures (DSS) were shown to occur around sites with 15–20 base pairs of near-identical sequence shared between recombination partners. Claessens et al. [4] confirm that these *var*-specific microhomology sharing recombinogenic sequences are ubiquitous throughout their much larger sample of crossovers. An analogy can be drawn with variable, diverse, and joining gene segment fusion (V[D]J)-based generation of diversity via recombination in the genes encoding immunoglobulins, although a similar pathway of error-prone nonhomologous end joining is not thought to be present in *P. falciparum*. Given the frame-conserving nature of *var* recombination, it seems most likely that accurate, microhomology-based homologous recombination repairs the double strand breaks occurring at *var* genes, within the DSS-marked recombination hotspots.

## Finishing off Malaria?

This remarkable WGS effort on hundreds of clones derived from asexually-generated lineages provides compelling evidence that *var* gene recombination is mainly, but not exclusively, mitotic in origin. Sex must still advantageously reshuffle *var* repertoires. The obvious biological advantage to the malaria parasite of high frequency mitotic *var* recombination, which Claessens et al. [4] show occurs in no other *P. falciparum* gene family, is that it allows generation of novel and functional PfEMP1 protein antigens during the malaria infection itself. This is now testable in vivo. The key role of *var*-based antigenic variation in malaria pathology is further established. We now know that chronic malaria infections survive their encounters with the host immune system [17] while generating novel antigenic variants via mitotic recombination at a very high rate. A somewhat sobering perspective on the huge problem of eradicating chronic, but asymptomatic, malaria infections is added.
